# Changes in Residency Applicant Cancellation Patterns with Virtual Interviews: A Single-site Analysis

**DOI:** 10.5811/westjem.18487

**Published:** 2024-03-14

**Authors:** Meryll Bouldin, Carly Eastin, Rachael Freeze-Ramsey, Amanda Young, Meredith von Dohlen, Lauren Evans, Travis Eastin, Sarah Greenberger

**Affiliations:** * University of Arkansas for Medical Sciences, Department of Emergency Medicine, Little Rock, Arkansas; † University of Arkansas for Medical Sciences, Department of Pediatrics, Section of Emergency Medicine, Toxicology, and Pharmacology, Little Rock, Arkansas

## Abstract

**Background:**

Residency programs transitioned to primarily virtual interviews due to the COVID-19 pandemic. This shift raised questions regarding expectations and patterns of applicant cancellation timeliness. The purpose of this study was to examine changes in applicant cancellations after transitioning to virtual interviews.

**Methods:**

This was a retrospective cohort study of interview data from a three-year emergency medicine residency at a tertiary-care academic medical center. Using archived data from Interview Broker, we examined scheduling patterns between one in-person (2019–2020) and two virtual interview cohorts (2020–2021 and 2021–2022). Our outcomes were the overall cancellation rates relative to interview slots as well as the proportion of cancellations that occurred within 7 or 14 days of the interview date.

**Results:**

There were 453 interview slots and 568 applicants invited. Overall, applicants canceled 17.1% of scheduled interviews. Compared with in-person interviews, applicants canceled significantly fewer virtual interviews (in person: 40/128 (31.3%), virtual year 1: 22/178 (12.4%), virtual year 2: 15/143 (10.5%), *P* = 0.001). Conversely, applicants canceled significantly more virtual interviews within both the 14-day threshold (in person: 8/40 (20%), virtual year 1: 12/22 (55.5%), virtual year 2: 12/15 (80%), *P* < 0.001) and the 7-day threshold (in person: 0/40 (0%), virtual year 1: 3/22 (13.6%), virtual year 2: 4/15 (26.7%), *P* = 0.004).

**Conclusion:**

While limited, at our site, changing to a virtual interview format correlated with fewer cancellations overall. The proportion of cancellations within 14 days was much higher during virtual interview seasons, with most cancellations occurring during that time frame. Additional studies are needed to determine the effects of cancellation patterns on emergency medicine recruitment.

## INTRODUCTION

Historically, residency applicants traveled to US programs for in-person interviews. In 2020, the COVID-19 pandemic led the Coalition for Physician Accountability (COPA) to recommend that residency programs conduct only virtual interviews.^1^ Proponents of virtual interviews cited cost and safety as potential upsides, and applicants have reported overall satisfaction with virtual interviews and more advantages than barriers.^2^ ^–^ ^4^ However, programs have expressed continued doubts about some aspects of virtual recruitment.^2^

Even before the pandemic, there were no established rules across specialties regarding an acceptable timeframe for interview cancellations. For emergency medicine, the Emergency Medicine Resident Association (EMRA) recommended at least two weeks’ notice in their 2019 “EMRA and CORD Student Advising Guide.”^5^ In 2020, the first year of virtual interviews, email communication on the Council of Residency Directors in Emergency Medicine (CORD) listserv suggested that program directors’ acceptable cancellation thresholds ranged from 48 hours to 10 days prior to the interview date.^6^ Ultimately, CORD stated that seven days was recommended for applicants in a 2020 blog post about interviewing during the pandemic, while other publications still recommended two weeks.^7^ ^,^ ^8^ Currently, the 2023 CORD Application Process Improvement Committee and the 2022–2023 National Resident Matching Program (NRMP) agreement have advised applicants to cancel no later than 1–2 weeks before their interview dates.^9^ ^,^ ^10^

Virtual interviews may be here to stay, as evidenced by recent COPA and Association of American Medical Colleges (AAMC) statements, as well as the 2023-24 CORD guidelines.^11^ ^–^ ^13^ Understanding patterns of virtual interview cancellation behavior may help program directors, applicants, and their advisors prepare for a successful Match. To characterize the effects of virtual recruitment on interview cancellations, we compared in-person interview cancellation patterns to those of virtual recruitment cycles at our academic emergency medicine (EM) residency.

## METHODS

This was a retrospective cohort study at a three-year EM residency sponsored by a tertiary-care, academic medical center in an urban setting in the south-central United States. This residency is an established program (founded in 1984) with a class size of 10 residents per year, which increased to 12 residents for the 2022 Match. The University of Arkansas for Medical Sciences Institutional Review Board (IRB) approved this study in exempt status.

Our program began using the online interview scheduling software Interview Broker (The Tenth Nerve, LLC, Lewes, DE; www.interviewbroker.com) in Fall 2019 to invite applicants to interview. In Fall 2020, interviews transitioned from in person to virtual and additional slots were added, with CORD continuing to recommend virtual interviews for EM residencies in subsequent cycles. Similar to in-person interviews, applicants for virtual interviews are invited in a 1:1 applicant to slot ratio and given 48 hours to respond before another applicant is invited.

Using archived data from Interview Broker, we examined scheduling patterns between the in-person interview cohort (2019–2020 season) and two virtual interview cohorts (Virtual Year 1: 2020–2021 and Virtual Year 2: 2021–2022). Unfortunately, cancellation data prior to the initiation of Interview Broker at our site was not available. A single investigator abstracted data from Interview Broker in aggregate form by academic year using overall counts of relevant variables, including number of interview slots, days, invitations, interviews scheduled/unscheduled (ie, no applicant response received)/declined, cancellations, and the timing of those cancellations relative to the interview date. We defined an interview cancellation as an interview that was scheduled, canceled, and never rescheduled; interviews that were rescheduled were considered completed. Demographic variables were not available as Interview Broker only records the student’s name and AAMC ID; accessing additional information would have required querying the Electronic Residency Application Service, which was not covered in our exempt IRB agreement.

Our outcomes were the overall proportion of interview cancellations relative to interview slots, as well as the proportion of interview cancellations that occurred within 14 days of the interview date and within seven days of the interview date. Descriptive statistics were performed. We performed comparisons using chi-squared or the Fisher exact test as some observations were uncommon. All comparisons were two-sided with ɑ = 0.05. Analyses were performed using SPSS Statistics for Macintosh Version 28.0 (IBM Corporation, Armonk, NY).

## RESULTS

Over three years, there were 453 interview slots and 568 applicants invited. Most of the interview slots were virtual (71.7%). Overall, the program sent out 1.25 interview applications per slot and applicants canceled 17.1% of scheduled interviews (Table 1). We found a significant decrease in the proportion of overall cancellations relative to filled interview slots, with 40/128 (31.3%), 22/178 (12.4%), and 15/143 (10.5%) cancellations for in-person, virtual year 1, and virtual year 2, respectively (*P* < 0.001). When analyzed further and adjusting for multiple comparisons, the decrease was significant when comparing in person vs. either virtual year, but not when comparing the two virtual years.

**Figure 1.**
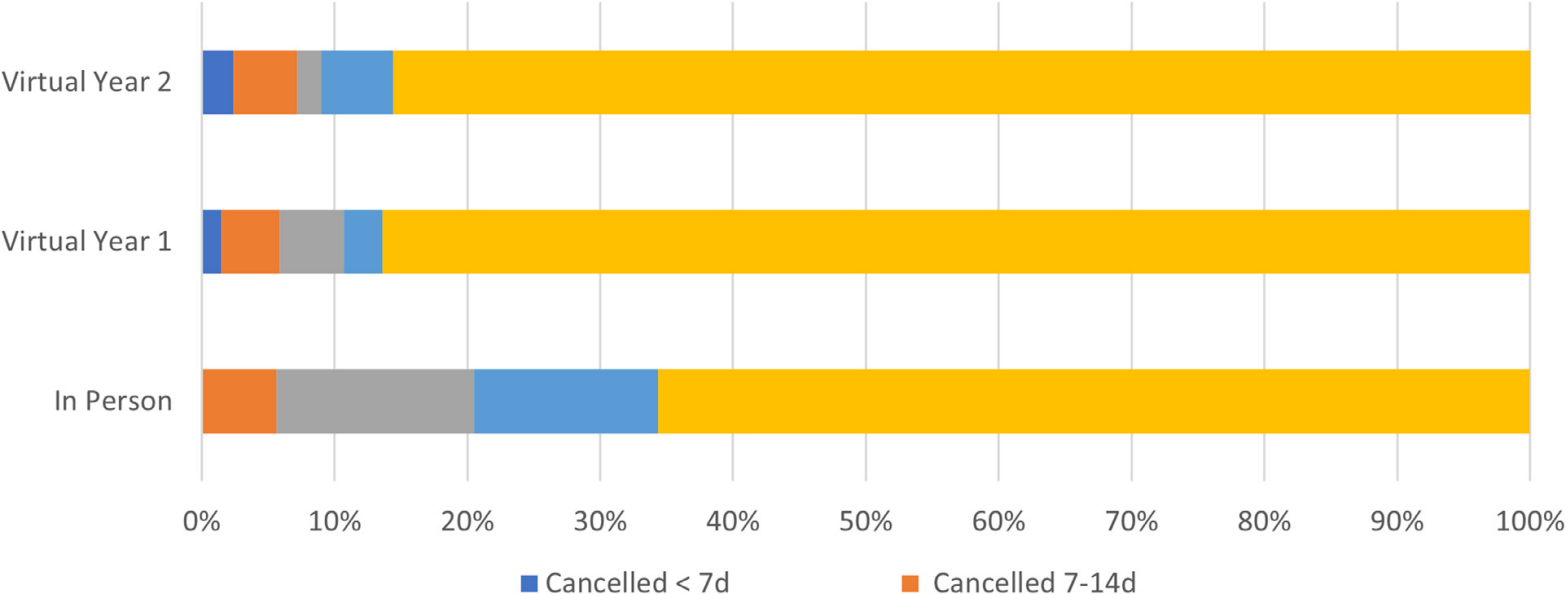
Overall distribution of invited applicants.

**Figure 2.**
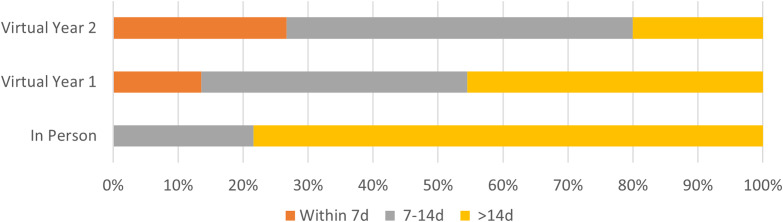
Interview cancellations by length of time from interview. *d*, day.

While fewer interviews were canceled, the proportion of virtual interview cancellations that occurred within 14 days of the interview date was significantly higher (in person: 8/40 (20%), virtual year 1: 12/22 (55.5%), virtual year 2: 12/15 (80%), *P* < 0.001). Similarly, more virtual interviews were canceled within seven days of the interview date (in person: 0/40 (0%), virtual year 1: 3/22 (13.6%), virtual year 2: 4/15 (26.7%), *P* = 0.004), although these numbers were low overall. In both the 14 and 7 day cancellation analyses, these data indicated a year-over-year increase, meaning in both 14 and 7 day comparisons we saw a significant increase in cancellations between in person and virtual year 1, and again saw a significant increase between virtual year 1 and virtual year 2. See Figures 1 and 2 for graphical breakdown of the overall distribution of invited applicants and interview cancellation rates.

**Table 1.** Breakdown of in-person and virtual interview cohorts; total counts provided unless otherwise specified.

Interviews and Cancellations			
Interview group	In person	Virtual year 1	Virtual year 2
Number of interview days	15	16	16
Number of interview slots	128	180	145
Number of applicants invited	195	206	167
Number of invitations per interview slot	1.52	1.14	1.15
Total interview slots filled	128	178	143
Number of unscheduled invitations (ie, no applicant response received)	14	1	3
Number who declined without scheduling	13	5	6
Overall cancellations (% of scheduled)	40 (31.3%)	22 (12.4%)	15 (10.5%)
Number who canceled < 7 days (% of canceled)	0 (0%)	3 (13.6%)	4 (26.7%)
Number who canceled 7–14 days (% of canceled)	8 (20%)	9 (40.9%)	8 (53.3%)
Number who canceled >14 days (% of canceled)	32 (80%)	10 (45.5%)	3 (20.0%)
Overall declined, unscheduled, or canceled (% of total invited)	67 (34.4%)	28 (13.6%)	24 (14.3%)

## DISCUSSION

Compared with in-person interviews, applicants to our program were less likely to cancel their virtual interview. Of those who did cancel, several virtual applicants canceled within seven days, and most cancellations occurred within 14 days of the interview date. For in-person interviews, applicants were traditionally instructed to cancel as soon as possible and at least two weeks prior to the interview date.^5^ As discussed previously, recommendations for EM virtual interview cancellations have ranged from 48 hours to two weeks, with the NRMP currently recommending at least 1–2 weeks in advance.^10^ Our results suggest that short-notice cancellations (ie, less than two weeks) by students may be more common in the virtual era.

We are not aware of literature regarding the specific timing of virtual interview cancellations, but our finding of fewer overall cancellations is consistent with Lewkowitz et al’s findings that maternal-fetal medicine fellowship virtual interviews had a lower rate of cancellations compared with in-person interviews (39.1% vs 72.3%).^14^ This could stem from the reduced time and cost required to interview virtually.^15^ ^,^ ^16^

Unfortunately, fewer interview cancellations overall could contribute to interview hoarding and an inequitable distribution of interviews. The AAMC and some specialties have expressed concerns about higher quality applicants receiving invitations for and scheduling excessively high numbers of interviews and leaving lower tier students with fewer options.^15^ ^,^ ^17^ While this has not been studied in EM specifically, the Emergency Medicine Consensus Statement on the 2020–2021 Residency Application process suggested an interview limit of 17 interviews and encouraged applicants not to interview at their less-preferred programs lower on their list to “make these slots available to other students,” indicating a potential concern for the effects of hoarding such as “peers not matching and/or programs not filling.”^18^

Short-notice interview cancellations pose a few other challenges for residency programs. Previously, filling an in-person interview slot required finding a replacement who could still arrange travel to the interview location, which is no longer relevant for virtual interviews. Nonetheless, the NRMP requires that programs provide no less than 48 hours for applicants to respond to interview invitations.^10^ If applicants are canceling only a few days before an interview, filling the open spot may be a challenge since programs cannot invite more than one applicant at a time per spot. Short-notice cancellations can also be problematic as interviewers may have to review candidates’ applications well in advance of the interview date. With short-notice cancellations, this could mean lost time for interviewers who had already reviewed those applications or inadequate time to review the replacements.

Conversely, program directors want to avoid interviewing applicants who are not interested in their program, and a cancellation—even on short notice— provides an opportunity to interview an applicant with greater interest in the program. In our case, we had only four open interview spots over the first two virtual years (two unfilled per year), indicating that we filled most canceled spots. Therefore, while no official opinion exists, program directors may not mind short-notice cancellation as long as the interview schedule is full. In fact, they may prefer for the applicant not to feel pressured to interview at a program in which they are uninterested only because they are concerned about canceling, with short notice being viewed as unprofessional. As virtual interviews appear to be here to stay, understanding cancellation patterns will be important for programs, especially in balancing the timing cancellations with new invitations so programs can ideally maintain a full interview schedule.

## LIMITATIONS

This study was limited to one specialty at a single institution, therefore the generalizabilty of these findings to other institutions or specialties is unclear, especially given the small sample size and limited pre-post period. The changing landscape of EM residency recruitment may also affect the generalizability of these findings. Unfortunately, we had only one year of in-person interview data as we did not keep these records prior to the use of Interview Broker, which could have introduced bias. We also had an increase in resident complement during virtual year 2, which may have confounded the results. Unfortunately, we were unable to include demographic data, which might have helped to identify additional cancellation patterns. Lastly, examining trends in those who reschedule interviews was not performed in this study and may be of value in future investigations, as some downsides discussed with short-notice cancellations (eg, filling empty slots; having time to review applications) would still occur in applicants who are rescheduling with short notice.

## CONCLUSION

Compared with in-person interview cycles, applicants to our residency program were significantly less likely to cancel virtual interviews. However, the majority of virtual cancellations that did occur were within 14 days of the interview date and nearly one-fifth occurred in under seven days. Additional studies, ideally multisite that include applicant demographic data, are needed to determine how cancellation patterns affect EM recruitment and match outcomes in the virtual era.
